# Supplementary material for: The InSight HP3 Penetrator (Mole) on Mars: Soil Properties Derived from the Penetration Attempts and Related Activities
Source: Space Sci Rev. 2022 Dec 9;218(8):72. doi: 10.1007/s11214-022-00941-z (PMC9734249; doi:10.1007/s11214-022-00941-z)
Supplement: Supplementary file 1 — (PDF 221 kB) [file 11214_2022_941_MOESM1_ESM.pdf]

Supplementary Material

Sol	Date	Phase	IDA	IDC	ICC	SEIS	STATIL	TEM-A	HAM	Description
	**2019**									
92	Feb 28	IA	x	x	x	x	x		3881	First penetration attempt, stereo pair (16:30)
94	Mar 02		x	x	x	x	x		4720	Second penetration attempt, stereo pair (17:00)
97	Mar 04	D&L						x		Conductivity measurement, 24 hours @ 1.0 W
98	Mar 05		x	x	x					3x6 mosaic (16:00), Sunset long-shadow, 'mole exit' pose 1
99	Mar 06		x	x	x					Sunrise long-shadow, 'mole exit' pose 1
103	Mar 10		x	x	x					SS window imaging, 'window' pose 1
106	Mar 13			x			x			Deck mosaic (debris check); Tilt measurement
107	Mar 14		x	x	x					Sunrise long-shadow, 'mole exit' pose 2
111	Mar 18		x	x	x					Sunset long-shadow, 'mole exit' pose 2
116	Mar 23							x		Conductivity measurement, 24 hours @ 2.0 W
118	Mar 25		x	x	x	x	x		197	Diagnostic Hammering 1 – IDC 'overhead' pose
127	Apr 03			x	x					SS window imaging, 'window' pose 2
128	Apr 04		x	x	x					2x2x1 mosaic (16:30)
146	Apr 23		x	x	x	x	x		-	*Diagnostic Hammering – did not execute*
158	May 05		x	x	x	x	x		198	Diagnostic Hammering 2, 'window' pose 2
203	Jun 22		x	x	x		x			12 cm lift to expose mole; no TLM reading (as expected)
206	Jun 25		x	x	x		x			13 cm lift (to 25 cm) to engage TLM
209	Jun 29		x	x	x		x			29 cm lift (to 54 cm) to extract ST and place SS
211	Jul 01							x		Conductivity measurement, 24 hours @ 2.0 W
227	Jul 17	PC	x	x	x					4x4x1 mosaic (12:00); ET imaging; 2x6 mosaic (17:30)
230	Jul 20		x	x	x					4x4x1 (12:00 & 16:00); 3x6 mosaic (17:00); pit closeup
240	Jul 30	RI-1	x	x	x	x	x			Flat scoop push; 4x4x1 mosaic (16:00)

243	Aug 03		x	x	x	x	x			1 scoop tip chop; 2x2x1 mosaic (14:40)<br>1 scoop tip chop; 2x2x1 mosaic (16:00)
246	Aug 05		x	x	x	x	x			1 scoop tip chop - *no ground contact*; 2x2x1 mosaic (14:40)<br>1 scoop tip chop - *no ground contact*; 2x2x1 mosaic (16:00)
250	Aug 08		x	x	x	x	x			1 scoop tip chop; 2x2x1 mosaic (14:40)<br>1 scoop tip chop; 2x2x1 mosaic (16:00)
253	Aug 12		x	x	x	x	x			1 scoop tip chop; 2x2x1 mosaic (14:40)<br>1 flat scoop push; 2x2x1 mosaic (16:00)
254	Aug 13		x	x	x	x	x			pit imaging
257	Aug 15		x	x	x	x	x			4x4x1 mosaic (16:00) – context
**Conjunction: 264 – 288**										
298	Sep 28		x	x	x					2x2x1 mosaic (16:00)
302	Oct 02	P1	x	x	x	x	x			Horizontal pinning
305	Oct 05		x	x	x	x	x			Vertical pinning
308	Oct 09			x	x	x	x		20	Hammer
311	Oct 12		x	x	x	x	x		101	Vertical pin (5 mm) + hammer
315	Oct 16		x	x	x	x	x		101	Vertical pin (5 mm) + hammer
318	Oct 19			x	x	x	x		152	Hammer
322	Oct 22	**REV1**	x	x	x	x	x		50	Slight retract IDA, horizontal move, regolith push + hammer
325	Oct 26		x	x	x	x	x		304	Hammer (150) + retract & push + hammer (150) → **Reversal**
329	Oct 29		x	x	x		x			IDA retract (2 cm horizontal; 2 cm vertical)
332	Nov 02		x	x	x					4x4x1 (13:00); 2x2x1 (16:00); pit closeup
339	Nov 09	P2	x	x	x		x			Horizontal pinning
342	Nov 12		x	x	x		x			Vertical pinning (1.5 cm down)
346	Nov 17			x	x	x	x		40	Hammer
349	Nov 19		x	x	x	x	x		50	Vertical pinning (4 cm down) + hammer
366	Dec 07			x	x	x	x		19	Hammer
373	Dec 14			x	x	x	x		127	Hammer
380	Dec 21			x	x	x	x	x	126	Hammer; Conductivity: 24 hours @ 2.0 W
	**2020**									
400	Jan 11	**REV2**	x	x	x	x	x			Vertical retract (1 cm up) + repining (3 cm down)
407	Jan 18			x	x	x	x		151	Hammer (150) → **Reversal**
414	Jan 25	RI-2	x	x	x		x			Horizontal offload (37.5 cm)
417	Jan 29		x	x	x		x			Vertical retract; 2x2x1 (15:00); scrape test; 2x2x1 (16:00)
420	Feb 01		x	x	x	x	x			Chop test; image; 2x2x1 mosaic (16:00)

454	Mar 07	BCP-H	x	x	x	?	x			Back cap preload – horizontal scoop (BCP-H) 01; 28 mm
458	Mar 11			x	x	x	x		24	Back cap hammer – horizontal scoop (BCH-H) 01
461	Mar 14		x	x	x					Re-applied preload on back cap
468	Mar 21		x	x	x	x	x			BCP-H 02 28 mm
472	Mar 25			x	x	x	x		24	BCH-H 02
482	Apr 04		x	x	x	x	x			BCP-H 03 18 mm
489	Apr 11			x	x	x	x		50	BCH-H 03
502	Apr 25		x	x	x	x	x			BCP-H 04 18 mm
509	May 02			x	x	x	x		100	BCH-H 04
516	May 09		x	x	x	x	x			BCP-H 05 18 mm
523	May 16			x	x	x	x		100	BCH-H 05
530	May 23		x	x	x	x	x			BCP-H 06 28 mm
536	May 30			x	x	x	x	x	151	BCH-H 06; Conductivity: 24 hours @ 2.0 W
543	Jun 06		x	x	x	x	x		100	BCP-H 07 32 mm; BCH-H 07
550	Jun 13		x	x	x	x	x		126	BCP-H 08 32 mm; BCH-H 08
557	Jun 20		x	x	x	x	x		151	BCP-H 09 32 mm; BCH-H 09
577	Jul 11		x	x	x	x	x			Retract and 4x4x1 mosaic (15:00)
598	Aug 01	RI-3	x	x	x	x	x			12 cm scrape (far-to-near) regolith into pit; mole obscured
599	Aug 02		x	x	x					4x4x1 mosaic (15:30)
611	Aug 15	BCP-I	x	x	x	x	x			Back Cap Preload – inclined scoop (BCP-I) 01 30 mm
618	Aug 22			x	x	x	x		101	Back Cap Hammer – inclined scoop (BCH-I) 01
632	Sep 05		x	x	x	x	x		101	BCH-I 02
645	Sep 19		x	x	x	x	x		252	BCH-I 03
659	Oct 03		x	x	x	x				Retract IDA, single images
660	Oct 04		x	x	x					4x4x1 mosaic (16:00)
673	Oct 17	RI-4	x	x	x	x				Two 12 cm scrapes (far-to-near); stereo image
680	Oct 24							x		Conductivity: 24 hours @ 2.0 W
686	Oct 31		x	x	x	x				Tamp regolith; 4x4x1 mosaic (15:30)
700	Nov 14		x	x	x	x				One 12 cm scrape (far-to-near); single and stereo images
734	Dec 19		x	x	x					Reposition IDA; regolith preload
	**2021**									
754	Jan 09	FFMT	x	x	x	x	x		506	Final Free Mole Test
775	Jan 30		x	x	x					Retract IDA; 2x2x1 mosaic (15:30)
795	Feb 13							x		Conductivity: 24 hours @ 2.0 W

Supplementary Table. Events during the mole recovery actions between Sols 253 and 472 on Mars. Listed are the sols on Mars, the terrestrial dates and the phases as given in Table 2. The activities of the various components and instruments are indicated. IDA: instrument deployment arm, IDC: instrument deployment camera, ICC: instrument context camera, SEIS: seismometer, STATIL: static tilt sensors, TEM-A: thermal conductivity measurement sensors, HAM gives the number of hammer strokes.

Ancillary movements of the IDA (coarse and fine positioning, grapple stow / release / restow, and such have been left out of the table. Pinning motions and imaging-only activities have been retained
